# The impact of the COVID-19 outbreak on Chinese-listed tourism stocks

**DOI:** 10.1186/s40854-021-00240-6

**Published:** 2021-04-02

**Authors:** Wenmin Wu, Chien-Chiang Lee, Wenwu Xing, Shan-Ju Ho

**Affiliations:** 1 School of Economics and Management, Nanchang University, Nanchang, China; 2 Research Center of the Central China for Economic and Social Development, Nanchang University, Nanchang, China

**Keywords:** COVID-19, Tourism, Event study method, Stock market, China, G14, L83, G14

## Abstract

This research explored the effects of the coronavirus disease (COVID-19) outbreak on stock price movements of China’s tourism industry by using an event study method. The results showed that the crisis negatively impacted tourism sector stocks. Further quantile regression analyses supported the non-linear relationship between the government’s responses and stock returns. The results present that the resurgence of the virus in Beijing did bring about a short-term negative impact on the tourism industry. The empirical results can be used for future researchers to conduct a comparative study of cultural differences concerning government responses to the COVID-19.

## Introduction

The contribution of the tourism industry to the global economy in the past few decades has continued to increase and, for many countries, it has become the most active and fastest-growing sector of their economy (Agbola et al. 2020). Its importance is manifested in the fact that it helps increase revenues (Alam and Paramati 2016), creates jobs (Habibi 2017), eliminates poverty (Blake et al. 2008), develops infrastructure (Lee and Chang 2008), and promotes economic growth (Mariolis et al. 2020). The overall contribution of tourism to China’s gross domestic product (GDP) hit more than 11% in 2018, and its role as a driving force of the national economy is getting stronger (Liu and Han 2020). However, the tourism industry is highly fragile and extremely susceptible to external shocks (Demiralay and Kilincarslan 2019; Lee and Chen 2020; Sigala 2020), such as terrorist attacks, wars, natural disasters, economic recessions, nuclear threats, and disease outbreaks (Seraphin 2017; Giusti and Raya 2019).

According to a report from the United Nations World Tourism Organization, the tourism industry contributed 10.4% to global GDP in 2018; however, owing to travel bans in many countries around the world as a result of the novel 2019 coronavirus disease (COVID-19) outbreak, international tourism fell by 22% in the first quarter of 2020 and is likely to fall by 60–80% for the whole year.^1^ China reported the first COVID-19 case in Wuhan, leading to its GDP to decline by 6.8% in the first quarter of 2020 compared with the same period of 2019 according to data released by National Bureau of Statistics. The literature has since expanded from the tourism-growth nexus (i.e., Perles-Ribes et al. 2017; Santamaria and Filis 2019; Croes et al. 2021; Lee et al. 2021) to its current focus of the impact of COVID-19 on the tourism stock market. This research therefore used the event study method (ESM) to investigate the effect of the COVID-19 outbreak on China’s tourism stocks to provide a better understanding of its impact on China’s stock markets.

Owing to the Chinese Lunar New Year’s population movement in 2020, the COVID-19 pandemic was accelerated through social contact. The Ministry of Culture and Tourism of China (MCTC) issued a notice to suspend the business activities of tourism enterprises starting from January 26, 2020, which led to a semi-closure of the nation’s entire tourism industry for a period. While many researchers have been concerned about the influence of market uncertainty triggered by the COVID-19 pandemic on tourism stocks and travel-related stocks, this paper focused on government policy responses and their impact on the relationship between COVID-19 and China’s tourism stocks. At present, China has one of the most rapidly developing emerging stock markets in the world (Hong et al. 2020; Liu et al. 2020d; Lee and Wang 2021), and although many scholars have shown strong interest on its market, Chinese tourism firms have received scant attention (Jiang et al. 2020). Therefore, this paper filled the gap in the extant literature on government policy responses to China’s stock markets and identified whether such responses have a mitigating effect on the relationship between COVID-19 and tourism stocks.

This paper reports four findings. First, the COVID-19 pandemic had a negative impact on the stock returns of Chinese-listed tourism firms. Second, there was a non-linear effect between China’s tourism stock returns and government responses. Third, government actions had a positive effect on the stock returns at the high quantile of the abnormal returns (ARs), indicating the coronavirus-return nexus benefitted from government responses. Fourth, the resurgence of the virus in Beijing affected the tourism industry.

Section 2 analyzes the timeline of COVID-19 and its impacts. Section 3 discusses the methodology and data. Section 4 reports the empirical evidence. Section 5 offers further analysis. Section 6 gives a brief conclusion.

## Timeline of COVID-19 and its impacts

### Timeline of the Chinese government’s reactions to the COVID-19 outbreak

The COVID-19 outbreak is one of the most influential health crises of the twenty-first century (Zenker and Kock 2020). It has spread all over the world, affecting economic sectors and financial markets. As the first country affected by the epidemic, China has spared no effort to adopt prevention and control measures to fight the virus. This section briefly describes timeline of China’s reaction to the COVID-19 outbreak.^2^

At the end of December 2019, there appeared cases of pneumonia of an unknown cause in Wuhan, Hubei Province. As the number of people with this type of pneumonia continued to increase, the Wuhan Municipal Health Commission (WHMHC) issued an emergency notice to medical institutions in its jurisdiction on December 30, 2019 and ordered to properly treat such patients. On the next day, the National Health Commission (NHC) arranged and dispatched a working group and a team of experts to the city to better guide responses to the epidemic and conduct on-site investigations. On the same day, in an effort to better inform the public, the WHMHC posted a bulletin about the pneumonia outbreak on its official website, confirming 27 cases and recommending individuals to wear masks when they go outside. To formulate emergency measures for the epidemic, NHC established a special leading group on January 1, 2020. Starting from January 3, 2020, China began to regularly report the real-time situation of the epidemic outbreak to the World Health Organization (WHO) as well as relevant countries and regions. After several days of concerted efforts, on January 9, 2020 an expert group of NHC stated that a novel type of coronavirus was preliminarily determined to be the cause of the pneumonia in Wuhan. China subsequently shared the news with the WHO and, on January 20, Zhong Nanshan, an academician at the Chinese Academy of Engineering, said in an interview that the virus could easily spread among the public and appealed to individuals not to go to Wuhan unless for a vital reason.

As soon as the news came out, it quickly attracted widespread public attention. To help control the outbreak and ensure people’s safety and health, Wuhan Epidemic Prevention and Control Headquarters released a notice to temporarily close channels leaving the city on January 23, 2020. On January 30, 2020, the Director-General of the WHO declared that the outbreak of the epidemic constituted a public health emergency of international concern. In the following days, China continuously adopted prevention, control, and treatment measures and kept the outside world informed.

**Fig. 1:**
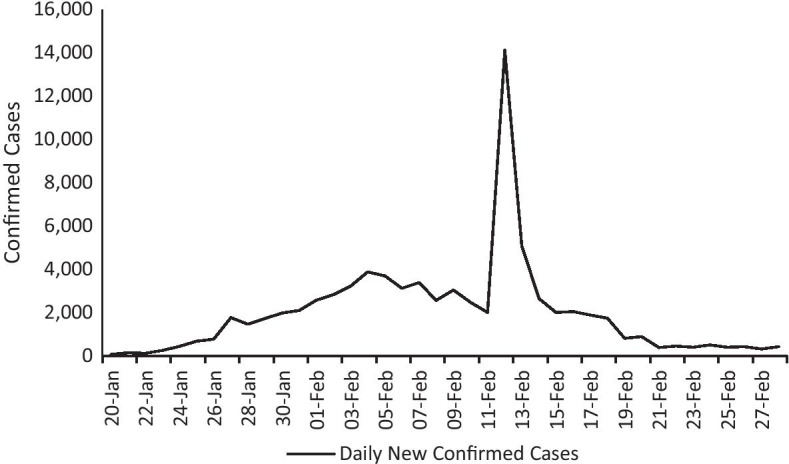
Daily new confirmed COVID-19 cases

To assist Hubei Province in overcoming this difficulty, other parts of China sent medical workers to the province. Through the joint efforts of a wide range of health and government staff, the epidemic in China was gradually controlled, as good news about the epidemic soon appeared. On February 17, the number of daily active confirmed cases nationwide was less than 2000 for the first time, and for the first time the data outside Hubei Province fell below 100, while the number of daily deaths across the country dropped below 100 for the first time as well. In March 17, there were no new local confirmed cases in cities outside Wuhan elsewhere in Hubei Province for 13 consecutive days. On March 19, a conference in Beijing announced that for the first time there were no new confirmed or suspected epidemic cases throughout China as of March 18. With the joint efforts of many individuals around the country, the country achieved outstanding results in fighting the epidemic, as shown in Fig. 1.

### Economic impacts

As the COVID-19 virus is highly contagious, many countries have adopted strict policies to restrict population movement, which caused a stagnation of economic activities to a large extent. Some scholars have studied the impact of COVID-19 on the economy. For example, Johns and Comfort (2020) noted that although the outbreak of COVID-19 has reduced greenhouse gas emissions, it also has caused serious economic and social problems, especially for developing countries. Djurovic et al. (2020) suggested that the government should adopt policies to stabilize the depressed economy of Montenegro owing to the impact of COVID-19. Ataguba (2020) noted that COVID-19 has brought significant economic losses for the economies of Africa.

Other researchers have focused on a specific industry that affects an economy following the COVID-19 outbreak. Chen et al. (2020) found that China’s offline consumption dropped by 32% following the COVID-19 outbreak, and the most affected sectors were catering, entertainment, and tourism. Furthermore, Austermann et al. (2020) addressed that the disruption of production and logistics caused by the epidemic outbreak has harshly hit China’s manufacturing sector. Haleem et al. (2020), Zenker and Kock (2020), and Altuntas and Gok (2021) stated that the direct impact on the economy and all manufacturing or hotel industries was from the quarantine measures and the closures of markets and factories. Sharma and Nicolau (2020) offered that tourism has especially experienced a substantial fall in valuation.

### Impacts on stock markets

COVID-19 has negatively affected investor sentiment that may have been amplified through social media, which has further impacted trading volume and stock prices for a sample of U.S. firms (Broadstock and Zhang 2019; Liu et al. 2020c). The impact of COVID-19 on stock markets has also been discussed in India and Australia (Mishra et al. 2020; Rahman et al. 2021). Liu et al. (2020a) found that COVID-19 has enhanced investors’ pessimistic judgments on future earnings and raised concerns about uncertainty. The number of daily new confirmed cases significantly negatively correlates with the returns of major stock indices, especially in Asia. He et al. (2020) used panel data to analyze the impact of the COVID-19 outbreak on the returns of Chinese-listed companies, indicating that its spread has had significantly adverse effects on the performance of domestic stocks across different industries. Using 75 countries as research samples, Erdem (2020) noted that the pandemic decreased stock returns and increased volatility. From a sample of 77 countries, Ashraf (2020b) indicated that compared with the growth of death cases, stock markets responded more proactively to confirmed cases. Topcu and Gulal (2020) studied the impact of the epidemic outbreak on emerging stock markets, showing that the adverse effect of the virus on these markets gradually diminished and started to phase out by mid-April.

As the COVID-19 pandemic first began to spread, tourism stocks and travel-related stocks were affected by market uncertainty. For example, the resultant oil price shock led to tourism stock prices falling. The reduction in supply (oil production has declined and oil prices have soared) caused tourism stock prices to fall (Shahzad and Caporin 2019; Narayan 2020; Qin et al. 2021). Tourism stock prices fell owing to a decline in consumer spending and corporate investment. COVID-19 initially hit consumer and business confidence, leading to a contraction in business investment decisions and personal consumption (Akron et al. 2020). In other words, businesses and consumers tended to reduce investment and postpone their decisions to wait for any uncertainty to disappear. Hence, in the face of information related to the COVID-19 outbreak, many investors did not partake in irrational investment behavior (Sun et al. 2021a). However, investor panic does have a negative impact on stock returns (Demiralay and Kilincarslan 2019; Wen et al. 2019; Aggarwal et al. 2020). The financial shock also caused tourism stock prices to fall. Many uncertainty factors made stock markets tumble and investment banks collapse (Caggiano et al. 2020; Sharif et al. 2020).

Recent literature on the effect of government responses to the COVID-19 pandemic remains sparse (Gonzalez-Bustamante 2021; Haldar and Sethi 2020; Miao et al. 2021). These papers discussed the effect of government intervention by improving stock liquidity (Haroon and Rizvi 2020; Carlitz and Makhura 2021) and alleviating the herding behavior of investors (Kou et al. 2019; Hong et al. 2020). We proposed the following two hypotheses:

#### **Hypothesis 1:**

The COVID-19 pandemic has a negative impact on tourism stocks.

#### **Hypothesis 2:**

The government response reduces the negative effect of the COVID-19 pandemic on tourism stocks.

## Methodology and sample collection

### Methodology

#### ESM

This research employed the ESM to analyze the impact of this global public health event on the stock price movements in China’s tourism sector. ESM has been widely applied to evaluate the impact of an event on stock markets. Nikkinen et al. (2008) studied the impact of 9/11 on returns and volatility of global stock markets, finding that the terrorist attack had a short-term negative impact on stock returns, and that the attack significantly increased volatility. Tee and Tessema (2018) analyzed stock market reactions to dividend announcements. Capelle-Blancard and Laguna (2010) selected 64 chemical industry explosion incidents worldwide from 1990 to 2005 as the research object, finding that the market values of the firms involved in those incidents decreased rapidly over the two days following the disaster, and that the loss in market value positively correlated with the number of deaths. Bash and Alsaifi (2019) presented that Jamal Khashoggi’s disappearance had an adverse effect on the earnings of stocks in the Kingdom of Saudi Arabia market. Articles have also applied ESM to test the effects of public health events on stock markets. Chen et al. (2007) explored the impact of severe acute respiratory syndrome (SARS) on the stock price movements of hotels in Taiwan, showing that the SARS outbreak adversely affected their earnings. Chen et al. (2009) presented that SARS brought negative impacts upon tourism, wholesale, and retail sectors; however, it had a positive impact on the biotechnology industry.

We therefore utilized the ESM to investigate the effects of the COVID-19 epidemic on the stock returns of Chinese-listed tourism firms. The ESM includes several steps, such as defining the event day, determining the event window as well as estimation window, selecting samples, calculating ARs and cumulative ARs (CARs), and testing their significance.

#### Event study set-up

Although there were confirmed COVID-19 cases in late December 2019, it was not until January 20, 2020, when Zhong Nanshan said that the virus could be spread among individuals, that the epidemic attracted wider public attention, and reports about the new pneumonia began to be reported in the media on a large scale. Therefore, we chose January 20, 2020, as the event day (t = 0).^3^ Consistent with Capelle-Blancard and Laguna (2010), our estimation period included 181 days— from March 28, 2019 to December 20, 2019—while (− 10,90) was the event window. To estimate ARs and CARs, we first computed expected returns (ERs). The market model is the most frequently used expected return model (Buigut and Kapar 2020):

1 $${R}_{i,t}={\alpha }_{i}+{\beta }_{i}{R}_{mt}+{\varepsilon }_{i,t}$$

Here, ${R}_{i,t}$ is the return of stock i at time t; ${R}_{mt}$ is the market return (we use the returns of SHCI and SZCI to represent market return in this paper) at time t within the estimated window; and ${\alpha }_{i}$ and ${\beta }_{i}$ are the coefficients to be estimated.

We calculate the measurement of these returns ${R}_{i}$ as follows:

2 $${R}_{i,t}=Ln\left({P}_{i,t}/{P}_{i,t-1}\right)$$

Here, ${P}_{i,t}$ and ${P}_{i,t-1}$ are the closing prices of firm $i$ on days $t$ and $t-1$, respectively. The ERs $E\left({R}_{i,t}\right)$ and ARs are then taken as

3 $$E\left({R}_{i,t}\right)={\widehat{\alpha }}_{i}+{\widehat{\beta }}_{i}{R}_{mt}$$

4 $${AR}_{i,t}={R}_{i,t}-E({R}_{i,t})$$

The AARs of the sample stocks on day t are computed as follows:

5 $${AAR}_{t}=\frac{{\sum }_{I=1}^{N}{AR}_{i,t}}{N}$$

Here, t represents time in the event window, and N is the total number of sample firms. We summed the individual ARs to get CARs. Similarly, we calculate the CAARs as follows:

6 $${CAR}_{i}\left({t}_{1},{t}_{2}\right)={\sum }_{t={t}_{1}}^{{t}_{2}}{AR}_{i,t}$$

7 $$CAAR\left({t}_{1},{t}_{2}\right)={\sum }_{t={t}_{1}}^{{t}_{2}}{AAR}_{t}$$

Here, ${t}_{1}$ and ${t}_{2}$ belong to the event window. We conducted t-tests for the significance of the results in this paper.

#### Regression analysis^4^

Following Al-Awadhi et al. (2020), we set the following model to test the impact of government response on stock returns:

8 $${AR}_{i,t}={\alpha }_{0}+{\alpha }_{1}{GRI}_{i,t-1}+\beta {Control}_{i,t-1}+{\varepsilon }_{i,t}$$

Here, ${AR}_{i,t}$ is the daily ARs discussed in the previous section. ${GRI}_{i,t-1}$ is the government response index (GRI) from the OxCGRT database.^5^${Control}_{i,t-1}$ denotes a series of control variables. Following Al-Awadhi et al. (2020), Ashraf (2020a, 2020b), and Zaremba et al. (2020), we selected the following control variables: daily growth rate of COVID-19 confirmed cases (${COVID-19}_{i,t-1}$), logarithm of market capitalization (${Log(macp)}_{i,t-1}$), and price-to-book ratio (${PB}_{i,t-1}$). The data of market capitalization and price-to-book ratio were obtained from the WIND Economic database. The daily confirmed cases of COVID-19 were obtained from the NHC of China.

The traditional regression model mainly examines the influence of explanatory variables on the conditional mean of the explained variables, and its description of the explained variables is not comprehensive. When there are outliers, collinearity, heteroscedasticity, etc., the results of an ordinary least squares (OLS) regression analysis may be biased. To make up for the shortcomings of traditional models, Demiralay and Kilincarslan (2019) used the method of quantile regression analysis, which can more comprehensively describe the behavior of explained variables, to analyze the relationship between explanatory variables and explained variables at different quantiles. In addition, it is less susceptible to heteroscedasticity and outliers than OLS and can provide more accurate empirical results.^6^ Inherent heterogeneity is also often higher under volatile market situations, and the relationship between market returns and independent variables may differ across their conditional distribution.

This research therefore employed quantile regression for analysis. Although the government policies caused a serious impact on society and the economy, they also did control the spread of the epidemic to a large extent, so that people’s lives could get back to normal as soon as possible. Gormsen and Koijen (2020) believed that these policies caused investors’ short- and long-term expectations to be inconsistent. Using a sample of 77 countries, Ashraf (2020b) found that although the governments’ isolation measures did have a direct negative impact on stock returns, the government’s income support plan also had a positive impact on the stock market to a large extent. Following Lee and Chen (2020), this research employed quantile regression analyses to explore the impact of government responses on China’s tourism stock returns. Specifically, we built the following quantile model:

9 $${Q}_{\tau }\left({AR}_{i,t}\right)={\alpha }_{0}^{\tau }+{\alpha }_{1}^{\tau }{GRI}_{i,t-1}+{\beta }^{\tau }{Control}_{i,t-1}+{\varepsilon }_{i,t}^{\tau }$$

Here, ${Q}_{\tau }\left({AR}_{i,t}\right)$ indicates the $\tau$th of the ARs, and ${\alpha }_{1}^{\tau }$ denotes the impact of GRI at the $\tau$th conditional quantiles of ARs. Consistent with most studies, we chose the five quantiles of 0.1, 0.25, 0.5, 0.75, and 0.9.^7^

### Sample collection

The tourism sector is a wide-ranging industry (Lori and Ashley 2018; Lee et al. 2021) that involves many aspects, such as food, hospitality, travel, visiting, shopping, and entertaining. To comprehensively analyze the impact of the COVID-19 epidemic outbreak on Chinese-listed tourism stock price movements, we followed the Wind Industry Classification Standards and chose tourism-related stocks (including airlines; marine; road and rail; and hotels, restaurants, and leisure) in its three-tier industries as the samples. For reliability, we eliminated stocks that were listed for less than three years or had been suspended multiple times during the event window. We thus selected 69 stocks: 9 airlines; 10 marine; 15 road and rail; and 35 hotels, restaurants, and leisure. We collected the daily closing prices of these 69 stocks as well as the Shanghai Composite Index (SHCI) and Shenzhen Composite Index (SZCI) from March 25, 2019 to July 10, 2020 on the China Stock Market and Accounting Research Database.

Table 1 presents the descriptive statistics and the summary statistics. The sample mean of ARs was negative after the official announcement of human-to-human transmission. The maximum and minimum values of the GRI indicated that the policies adopted by the government have changed. The mean of COVID-19 was 0.07491, indicating that COVID-19 had a 7.5% daily increase on average. The average of the logarithm of market capitalization (Logmcp) and the price-to-book ratio were 4.15 and 4.53, respectively.

**Table 1:** Summary statistics

Variable	Mean	SD	Min	Max
AR	− 0.00008	0.02541	− 0.14898	0.15466
GRI	61.64447	9.14847	16.67	68.45
COVID-19	0.07491	0.14797	0.00012	0.64541
Logmcp	4.145	1.26694	2.10967	7.43644
PB	5.53166	27.05623	− 7.7558	275.7954

AR is abnormal returns. GRI is the government response index obtained from Oxford COVID-19 Government Response Tracker. COVID-19 represents the daily growth rate of COVID-19 confirmed cases calculated as $\left(\left({Case}_{t}-{Case}_{t-1}\right)/{Case}_{t-1}\right)$. Logmacp is the logarithm of market capitalization. PB represents the price-to-book ratio

## Empirical results

### Results of AARs and CAARs

The implementation of epidemic prevention measures, such as community isolation, travel bans, stay-at-home sports/exercises, etc., has caused severe damage to global tourism and leisure activities (Sigala 2020). In this section we analyze whether the outbreak of the COVID-19 virus impacted the stock price movements of Chinese-listed tourism firms. Figure 1a and Table 2 present the results of AARs across all samples from days -10 to 30, showing that AARs gradually decline at least 4 trading days prior to the event day. For the 5 days after the event day, AARs are all negative, and there was a significant decline in returns. Thus, the COVID-19 epidemic had a significant adverse effect on China’s tourism stock market, which is in line with the findings of Sun et al. (2021b). This supports the first hypothesis.

**Table 2:** The results of AARs between days − 10 and 30

T	Number of firms	AARs	T-value
− 10	69	− 0.003551	− 1.573988
− 9	69	0.005680**	2.513957
− 8	69	− 0.002665	− 1.178140
− 7	69	0.002029	0.896718
− 6	69	0.001767	0.783321
− 5	69	− 0.007394***	− 3.270436
− 4	69	0.004301*	1.905923
− 3	69	− 0.003005	− 1.331611
− 2	69	− 0.002971	− 1.316587
− 1	69	− 0.002944	− 1.304893
0	69	− 0.013142***	− 5.814070
1	69	− 0.008092***	− 3.572430
2	69	− 0.005332**	− 2.361416
3	69	− 0.003969*	− 1.726461
4	69	− 0.017352***	− 6.765015
5	69	− 0.060462***	− 26.567270
6	69	0.004923**	2.171587
7	69	− 0.005568**	− 2.446362
8	69	0.009950***	4.409925
9	69	0.004093*	1.812128
10	69	0.001481	0.656327
11	69	− 0.000506	− 0.223525
12	69	− 0.003304	− 1.463081
13	69	− 0.004776**	− 2.116601
14	69	0.000672	0.294425
15	69	0.006897***	3.056579
16	69	0.013707***	6.073298
17	69	0.011830***	5.202713
18	69	− 0.007522***	− 3.331388
19	69	− 0.021499***	− 9.519257
20	69	− 0.010199***	− 4.517518
21	69	0.020883***	9.183845
22	69	0.007328***	3.247859
23	69	0.001119	0.478916
24	69	− 0.000443	− 0.192524
25	69	− 0.002137	− 0.946186
26	69	0.004573**	2.026268
27	69	0.002064	0.908412
28	69	0.009600***	4.243869
29	69	0.022222***	9.614587
30	69	− 0.001675	− 0.736128

*t* statistics in parentheses. *p < 0.1; **p < 0.05; ***p < 0.01

A public health event like the COVID-19 outbreak is considered a catastrophe; thus, investors would expect tourism companies to exhibit bad future performance and their stock prices to go down, thereby eventually reducing stock returns (Liu et al. 2020b). The highest negative return for the tourism sector is − 0.060462 on day 5 after the event day, in which T = 5 was the second day after the stock market re-opened following the Chinese Lunar New Year Festival. There are several possible reasons for the largest negative return on that day: (1) the number of daily new confirmed case increased rapidly during this holiday, (2) the Ministry of Culture and Tourism of China suspended the business activities of travel companies during this time, and (3) little was known about the virus at that time. All these reasons influenced investors’ negative views of the tourism industry. The results are also consistent with the recent finding of Chinese investors’ awareness of the dangers triggered by the COVID-19 pandemic in China’s stock markets (Corbet et al. 2020).

**Fig. 2:**
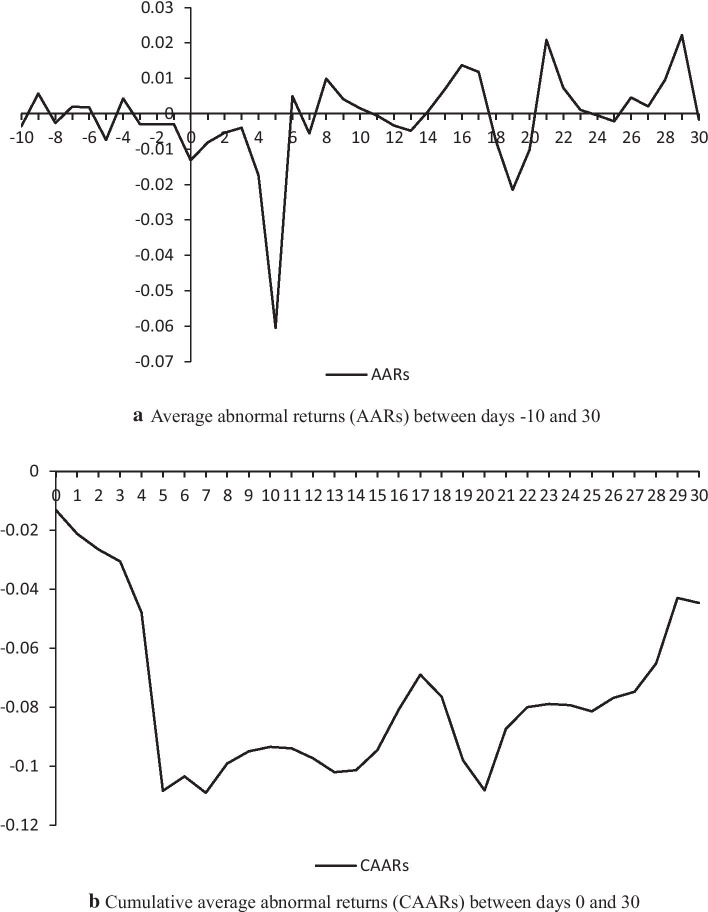
AARs and CAARs

Figure 2b provides CAARs across all firms from days 0 to 30. Table 3 reports CAARs of different event windows. As shown in Fig. 2b, there was a sharp decline in CAARs after the event; yet, in the short term, the CAARs did not change substantially. Simultaneously, the outbreak of COVID-19 brought negative ARs to most tourism firms. Beginning from that day, the number of firms experiencing negative ARs started increasing. From day 1 to day 20, the proportion of companies with negative CARs changed from 72 to 93%, indicating that most firms were negatively affected (Table 3). This result is similar to a recent outcome on the catering and lodging industries (Liu et al. 2020b). From Table 3, we concluded that although CAARs remained negative in the fourth month after the event, they were not significantly different from zero, which might be owing to stronger transparent and accessible information and effective control of the epidemic (Kou et al. 2014; Gu 2020; Wang et al. 2020), people’s lives gradually returning to normal as usual, and tourism gradually recovering.

**Table 3:** The results of CAARs for different event windows

Event window	CAAR	T-value	SD	CAR < 0 (%)
(0,0)	− 0.01314***	− 5.749077	0.033248	72
(0,1)	− 0.021234***	− 6.579031	0.047299	72
(0,2)	− 0.026566***	− 6.721064	0.052517	81
(0,3)	− 0.030536***	− 6.661685	0.056360	75
(0,4)	− 0.047888***	− 8.923224	0.061830	84
(0,5)	− 0.108350***	− 18.814188	0.071108	94
(0,6)	− 0.103427***	− 16.817030	0.070520	94
(0,7)	− 0.108995***	− 16.730179	0.072718	96
(0,8)	− 0.099045***	− 14.357809	0.066615	96
(0,9)	− 0.094953***	− 13.087063	0.066889	91
(0,10)	− 0.093472***	− 12.297848	0.063869	94
(0,20)	− 0.108171***	− 10.345016	0.077732	93
(0,30)	− 0.044637***	− 3.507018	0.111051	80
(0,40)	0.014397	0.967282	0.162791	48
(0,50)	0.004404	0.264680	0.175272	52
(0,60)	− 0.004833	− 0.263091	0.193681	61
(0,70)	− 0.05501***	− 2.742423	0.170263	74
(0,80)	− 0.024909	− 1.136088	0.219950	58
(0,90)	− 0.004275	− 0.180390	0.253282	54

CAAR denotes the cumulative average abnormal returns. SD is the standard deviation. *t* statistics in parentheses. *p < 0.1; **p < 0.05; ***p < 0.01

### Panel regression results of the government responses and ARs

This section analyzes the relationship between a series of government responses to the epidemic and the returns of Chinese tourism stocks. As the virus is highly contagious, governments around the world have conducted many strategies to restrict public movement to protect life and health. For example, after Zhong Nanshan said on January 20 that the virus can spread among people, the China government announced on January 23 the closure of the channels out of Wuhan, and MCTC announced on January 26 that the business activities of tourism enterprises were suspended across the country. There is no doubt that these measures will greatly affect investors’ judgment on the future development of the tourism industry. Although a few scholars have analyzed the influence of government reaction on financial markets (see Haroon and Rizvi 2020; Narayan et al. 2020; Phan and Narayan 2020), as far as we know, this research was the first to analyze the impact of policies on the stock returns of a specific industry.

Table 4 columns (1) and (2) represent the results of panel regression. After controlling for firm-specific characteristics and the growth rate of COVID-19 confirmed cases, government responses were significantly negative with $ARs$, indicating that government policies brought an unequivocal negative impact on the stock returns of the domestic tourism sector. This is in line with the finding of Zaremba et al. (2020), who showed that government responses cause an additional impact on stock market volatility. This is also consistent with the second hypothesis.

**Table 4:** Panel regression results of the growth in COVID-19 confirmed cases and abnormal returns

Variable	(1)	(2)	(3)	(4)	(5)	(6)	(7)
	AR	AR	AR	AR	AR	AR	AR
Con	0.01062*** (3.31)	0.06563*** (2.69)	0.00527 (0.66)	− 0.00707* (− 1.82)	− 0.00250 (− 0.76)	0.00336 (0.63)	0.00536 (0.51)
GRI	− 0.00011** (− 2.40)	− 0.00014** (− 2.14)	− 0.00042*** (− 3.66)	− 0.00002 (− 0.28)	0.00006 (1.30)	0.00016** (2.14)	0.00044*** (2.90)
COVID-19	− 0.03794*** (− 9.67)	− 0.03891*** (− 6.60)	− 0.06745*** (− 6.24)	− 0.02006*** (− 3.83)	− 0.00888** (− 1.99)	− 0.00701 (− 0.97)	− 0.00035 (− 0.02)
Log(macp)	− 0.00041 (− 1.39)	− 0.01314** (− 2.32)	0.00042 (0.67)	− 0.00050* (− 1.65)	− 0.00046* (− 1.80)	− 0.00066 (− 1.57)	− 0.00128 (− 1.55)
PB	0.00003*** (9.51)	− 0.00002 (− 0.13)	0.00001 (0.47)	0.00001 (0.89)	0.00002** (1.98)	0.00009*** (4.53)	0.00006 (1.57)
Adj./Pseudo R^2^	0.0233	0.0258	0.0205	0.0076	0.0068	0.0135	0.0250
*N*	3312	3312	3312	3312	3312	3312	3312

*t* statistics in parentheses. *p < 0.1; **p < 0.05, and ***p < 0.01. AR denotes the abnormal returns. GRI is the government response index obtained from Oxford COVID-19 Government Response Tracker. COVID-19 represents the daily growth rate of COVID-19 confirmed cases calculated as $\left(\left({Case}_{t}-{Case}_{t-1}\right)/{Case}_{t-1}\right)$. Logmacp is the logarithm of market capitalization. PB represents the price-to-book ratio. Columns (1) and (2) show the results of pooled OLS and fixed-effects, respectively. Columns (3)–(7) represent the quantile regression results with five quantiles q = {0.1; 0.25; 0.5; 0.75, 0.9}

Table 4 columns (3)–(7) show the results of the quantile regression analysis, presenting the non-linear effects on China’s tourism stock returns and government responses. First, when the ARs were at the low quantile, government actions had a negative effect on stock returns, which is consistent with Ashraf (2020b). However, at upper quantiles, government intervention had a significantly positive impact on stock returns. This result is consistent with Narayan et al. (2020), who noted that government responses increase stock returns. In addition, for the high stock return quantile, the significant negative relationship between the growth rate of COVID-19 confirmed cases and stock returns disappears, which confirms the results of Haroon and Rizvi (2020), Narayan et al. (2020), and Topcu and Gulal (2020), who believed that policies increase liquidity in the stock market and dispel investors’ fears, thus offsetting the negative impact of the pandemic on stock returns.

There are several possible reasons for the non-linear correlations. First, at the low quantile, China is experiencing heavy virus infections. To control the spread of the epidemic, the government took a series of steps, such as closing bus routes and workplaces. Specifically, the suspension of business activities of travel agencies has had a great negative influence on the tourism industry. Second, at upper quantiles, the early measures taken by the government to control the spread of the epidemic achieved beneficial results. After the epidemic was effectively controlled, people trapped at home for a long time increased their demand for tourism, thus raising the cash flows of the tourism industry. Some income and debt support policies provided by the government in the later period also strengthened investors’ confidence as well. Those policies play a positive role in spurring the market’s response and weaken the negative relationship of confirmed cases with the stock market. To make the results more reliable, the Stringency Index was used as a proxy for GRI (Table 5).

**Table 5:** The results of the robustness tests

Variable	(1)	(2)	(3)	(4)	(5)	(6)	(7)
	AR	AR	AR	AR	AR	AR	AR
Con	0.00775*** (3.33)	0.06022** (2.52)	− 0.00654 (− 1.21)	− 0.00743*** (− 2.69)	− 0.00114 (− 0.49)	0.00604 (1.59)	0.01545** (2.09)
SI	− 0.00005** (− 2.20)	− 0.00007* (− 1.91)	− 0.00021*** (− 3.44)	− 0.00001 (− 0.29)	0.00004 (1.33)	0.00011** (2.46)	0.00025*** (2.92)
COVID-19	− 0.03536*** (− 10.20)	− 0.03567*** (− 7.05)	− 0.05426*** (− 6.16)	− 0.01985*** (− 4.41)	− 0.00976** (− 2.55)	− 0.00795 (− 1.28)	− 0.01005 (− 0.84)
Log(macp)	− 0.00040 (− 1.39)	− 0.01272** (− 2.25)	0.00053 (0.89)	− 0.00048 (− 1.59)	− 0.00048* (− 1.86)	− 0.00071* (− 1.70)	− 0.00133 (− 1.64)
PB	0.00003*** (9.51)	− 0.00002 (− 0.14)	0.00002 (0.56)	0.00001 (0.88)	0.00002** (1.98)	0.00009*** (4.55)	0.00006 (1.58)
Adj./Pseudo R^2^	0.0231	0.0255	0.0195	0.0076	0.0068	0.0136	0.0247
N	3312	3312	3312	3312	3312	3312	3312

*t* statistics in parentheses. *p < 0.1; **p < 0.05, and ***p < 0.01. AR denotes the abnormal returns. SI is the government response stringency index obtained from Oxford COVID-19 Government Response Tracker. COVID-19 represents the daily growth rate of COVID-19 confirmed cases calculated as $\left(\left({Case}_{t}-{Case}_{t-1}\right)/{Case}_{t-1}\right)$. Logmacp is the logarithm of market capitalization. PB represents the price-to-book ratio. Columns (1) and (2) show the results of pooled OLS and fixed-effects, respectively. Columns (3)-(7) represent the quantile regression results with five quantiles q = {0.1; 0.25; 0.5; 0.75, 0.9}

## Further analysis

With the joint efforts of many citizens and doctors in China, the COVID-19 epidemic in the country has effectively been controlled. Since the end of May 2020, the number of daily new confirmed local cases has been zero, people’s lives had gradually returned to normal, and the tourism market began gradually recovering from the epidemic. However, on June 11, 2020, the capital of Beijing reported a new confirmed local case of COVID-19, breaking the record of zero daily new confirmed cases. In the following days afterward, other confirmed cases began to appear. Therefore, we take this as the background to analyze whether the epidemic resurgence in Beijing impacted China’s tourism market again.

**Fig. 3:**
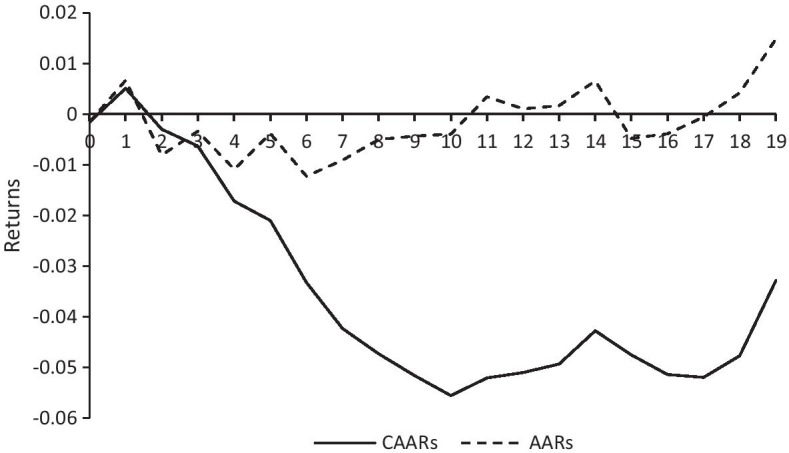
AARs and CAARs between days 0 and 19

We similarly used the ESM for analysis. June 11, when Beijing reported a new local confirmed case, was defined as the event day. For reliability, the estimation window of this event was the same as before. Figure 3 reports the results of AARs and CAARs between days 0 and 19.

Table 6 shows the values of AARs and CAARs after the virus came back in Beijing. Although the abnormal return was negative on the event day, it was non-significant, which may be owing to the fact that investors were not sure whether the news was true when it came out. After day 1, the ARs began to turn significantly negative, indicating that that tourism market was affected by the event, albeit with a delay. This may be owing to the fact that starting from June 11, there were new confirmed local cases in Beijing every day, which led to investors feeling pessimistic about the market and caused a decline in share prices. It also means that the resurgence of the epidemic in Beijing had a negative impact on China’s tourism sector again. Investors eventually decided that the epidemic in Beijing was minimal; so, stock prices began to rise. Starting from day 11, the AARs turned positive. This result is in line with Corbet et al. (2020) and Xiong et al. (2020), indicating that the response to the COVID-19 outbreak was stronger in industries that are vulnerable to its pandemic; while, in Beijing, which exhibits fast information liquidity, investors were aware of the pandemic and able to manage the risks.

**Table 6:** AARs and CAARs from days 0 to 19 of the epidemic resurgence in Beijing

t	CAARs (0, t)	AARs	CARs < 0 (%)
0	− 0.001567	− 0.001567	55
1	0.005127	0.006694***	46
2	− 0.002952	− 0.008079***	58
3	− 0.006295	− 0.003343	61
4	− 0.017161***	− 0.010866***	61
5	− 0.020954***	− 0.003794*	68
6	− 0.033174**	− 0.012220***	64
7	− 0.042277***	− 0.009103***	78
8	− 0.047280***	− 0.005002**	80
9	− 0.051588***	− 0.004309*	84
10	− 0.055520***	− 0.003931*	81
11	− 0.052063***	0.003456	83
12	− 0.050985***	0.001078	83
13	− 0.049305***	0.001680	81
14	− 0.042733**	0.006572***	78
15	− 0.047504**	− 0.004771**	81
16	− 0.051361***	− 0.003857*	84
17	− 0.051938**	− 0.000577	78
18	− 0.047702**	0.004236*	78
19	− 0.032866	0.014836***	77

CAARs represent the cumulative average abnormal returns. AARs denote the average abnormal returns. *t* statistics in parentheses. *p < 0.1; **p < 0.05; ***p < 0.01

Though both events had an adverse effect on China’s tourism market, compared with the first event, the rebound of the epidemic in Beijing had a smaller negative effect on the tourism market. On the one hand, in the first event, more than 90% of firms experienced negative returns; on the other hand, the largest negative CAARs of the first and latter event were − 0.108995 and − 0.055520, respectively.

## Conclusion

Our research employed an ESM to analyze the impact of the COVID-19 outbreak on China’s stock markets. We found that it had a negative effect on travel-related stocks. On this basis, this paper further analyzed government responses on the influence of COVID-19 on stock returns via quantile regression analyses. The results yielded a non-linear relationship between government intervention and ARs of Chinese tourism stocks. Our results also suggest the negative impact of COVID-19 on tourism stocks, where government intervention was present, which gradually decreased with the quantile level and was significantly positive during the highest ARs on tourism stocks. When the tourism stock market has ARs, the results showed that government intervention is effective and has a certain economic significance. Therefore, government interventions concerning stock prices amid the COVID-19 pandemic play important roles in alleviating its negative impact on tourism stocks, which may be partly related to the elimination of investors’ COVID-19-related fears. We further analyzed the impact of the resurgence of the epidemic in Beijing on the tourism industry. The outcomes verified the main findings, although the negative impact of the rebound on tourism stocks was only short-term.

Owing to the fact that the pandemic in China is still progressing, there are some limitations and possible future directions for conducting follow-up studies. One possible direction of research is to examine the impact of COVID-19 on China’s global supply chains from different economies and industries. By reflecting upon effective government intervention measures, researchers are recommended to consider corruption, long-/short-term policies, or other control factors. Along with government responses, researchers should also consider how to offset the costs faced by the various industries already affected, by providing a more comprehensive analysis of the impact of government action on other industries.

## Data Availability

The data that support the findings of this study are available upon request from the corresponding author.

## Footnotes

1 Please see the reference from the following website: https://www.unwto.org/news/covid-19-international-tourist-numbers-could-fall-60-80-in-2020.

2 Source: http://xinhuanet.com.

3 Sun et al. (2021b) used January 20, 2020 in their event study method.

4 This analysis was done by STATA 16; StataCorp LLC, Texas, USA.

5 Our database was obtained from https://www.bsg.ox.ac.uk/research/research-projects/coronavirus-government-response-tracker. This database has 14 sub-indices, including closing schools, workplace and public transport, cancelling public events, restricting on gathering size and internal movement or travel, staying at home, supporting income, giving debt/contract relief for households, promoting public information campaign, testing policy, tracing contact, and covering faces.

6 Please see Brooks (2013) for more information.

7 Five quantiles were used by those studies (Marrocu et al. 2015; Lin and Xu 2017; Xu et al. 2017; Lee and Chen 2021).
